# Loss of Biliverdin Reductase Increases Oxidative Stress in the Cyanobacterium *Synechococcus* sp. PCC 7002

**DOI:** 10.3390/microorganisms11102593

**Published:** 2023-10-20

**Authors:** Wendy M. Schluchter, Courtney H. Babin, Xindi Liu, Amori Bieller, Gaozhong Shen, Richard M. Alvey, Donald A. Bryant

**Affiliations:** 1 Department of Biological Sciences, University of New Orleans, New Orleans, LA 70148, USA; cchymel@my.uno.edu (C.H.B.); xliu9@my.uno.edu (X.L.); abiell3@lsu.edu (A.B.); 2 Department of Biochemistry and Molecular Biology, The Pennsylvania State University, University Park, PA 16802, USAralvey@iwu.edu (R.M.A.); dab14@psu.edu (D.A.B.); 3 Biology Department, Bloomington, Illinois Wesleyan University, Bloomington, IL 61702, USA

**Keywords:** antioxidant, biliverdin, biliverdin reductase, bilirubin, cyanobacteria, phycobiliproteins, photosynthesis, reactive oxygen species, redox cycle

## Abstract

Oxygenic photosynthesis requires metal-rich cofactors and electron-transfer components that can produce reactive oxygen species (ROS) that are highly toxic to cyanobacterial cells. Biliverdin reductase (BvdR) reduces biliverdin IXα to bilirubin, which is a potent scavenger of radicals and ROS. The enzyme is widespread in mammals but is also found in many cyanobacteria. We show that a previously described *bvdR* mutant of *Synechocystis* sp. PCC 6803 contained a secondary deletion mutation in the *cpcB* gene. The *bvdR* gene from *Synechococcus* sp. PCC 7002 was expressed in *Escherichia coli*, and recombinant BvdR was purified and shown to reduce biliverdin to bilirubin. The *bvdR* gene was successfully inactivated in *Synechococcus* sp. PCC 7002, a strain that is naturally much more tolerant of high light and ROS than *Synechocystis* sp. PCC 6803. The *bvdR* mutant strain, BR2, had lower total phycobiliprotein and chlorophyll levels than wild-type cells. As determined using whole-cell fluorescence at 77 K, the photosystem I levels were also lower than those in wild-type cells. The BR2 mutant had significantly higher ROS levels compared to wild-type cells after exposure to high light for 30 min. Together, these results suggest that bilirubin plays an important role as a scavenger for ROS in *Synechococcus* sp. PCC 7002. The oxidation of bilirubin by ROS could convert bilirubin to biliverdin IXα, and thus BvdR might be important for regenerating bilirubin. These results further suggest that BvdR is a key component of a scavenging cycle by which cyanobacteria protect themselves from the toxic ROS byproducts generated during oxygenic photosynthesis.

## 1. Introduction

Biliverdin reductase (BvdR; E.C. 1.3.1.24), which has been widely studied in mammals, catalyzes the conversion of the heme catabolic product, biliverdin IXα (Bvd), to bilirubin. Although Bvd is a water-soluble compound, bilirubin is poorly soluble in water because of intramolecular H-bonding. In mammals, bilirubin binds to albumin in blood and must be glucuronidated before being excreted in bile [1]. It is somewhat puzzling that mammals reduce Bvd because it is more complicated to eliminate bilirubin, and when not eliminated properly, bilirubin can cause jaundice in mammals [2]. However, a landmark study revealed that bilirubin is an important lipid-soluble antioxidant at physiologically relevant O_2_ concentrations and is apparently more important than α-tocopherol [3]. It was additionally shown that the albumin-bound form of bilirubin protects linoleic acid from oxidation by peroxyl radicals [4]. Even though bilirubin levels (20–50 nM) are low in tissues, one study showed that the addition of as little as 10 nM bilirubin protected brain cells from concentrations of peroxide more than four orders of magnitude higher [5]. Other scientists then hypothesized that, when bilirubin is oxidized to Bvd, BvdR ensures that it is converted back to bilirubin, creating a powerful redox cycle and explaining how low levels of bilirubin can afford protection against much higher concentrations of peroxide [6]. These studies also showed that bilirubin is important in protecting cells from reactive oxygen species (ROS), that this redox cycle can occur in vitro, and that Bvd is produced when bilirubin reacts with ROS [6]. RNAi studies showed that the depletion of BvdR led to increased sensitivity to oxidative stress and apoptosis, whereas the depletion of glutathione, previously thought to be the major protector of tissues against ROS, led to a much smaller increase in oxidative stress sensitivity. Both bilirubin and glutathione are probably important in cells. Glutathione is presumably more important in protecting cytoplasmic constituents, whereas the lipophilic bilirubin may play a more important role in preventing lipid peroxidation [6,7].

In mammals, bilirubin is mainly bound to albumin in the blood and has been shown to have antioxidant activity in this form [4]. There have been some challenges concerning the role of BvdR in an antioxidant cycle [8,9,10], but the consensus remains that bilirubin and Bvd play important roles in scavenging ROS in animals [11,12]. Mammalian BvdR is an autokinase and phosphorylation is required for the reduction of biliverdin to bilirubin [13]. BvdR is also involved in insulin and MAPK signaling as well as in acting as a transcription factor [14,15,16]. Therefore, in mammals, BvdR is an important component of a potent redox cycle that links heme metabolism, gene expression, and cell signaling. 

Schluchter and Glazer [17] reported that the cyanobacterium *Synechocystis* sp. PCC 6803 (hereafter *Synechocystis* 6803) has a functional BvdR. BvdR from *Synechocystis* 6803 is a polypeptide of 328 residues that can form dimers [18] or monomers [19] and has an acidic pH optimum for activity with NADPH [17]. The structure of monomeric BvdR has been solved using X-ray crystallography to a resolution of 2.1 Å and was surprisingly found to bind two Bvd molecules in the cyclohelical conformation in a nearly parallel, stacked manner in close proximity to one another (~4 Å). The proximal Bvd molecule binds in close proximity (~2.6 Å) to the nicotinamide moiety of NADP^+^ (Takao et al., 2017). Because the crystals retained catalytic activity, it is assumed that the structure represents that of the active enzyme. In cyanobacteria, BvdR lacks an obvious signal peptide for localization to the thylakoid lumen/periplasmic space of cyanobacteria, indicating that the enzyme is present in the cytoplasm. Surprisingly, a *bvdR* mutant of *Synechocystis* 6803 made no phycocyanin, a light-harvesting protein carrying the linear tetrapyrrole chromophore, phycocyanobilin, whose biosynthetic precursor is Bvd [17]. Schluchter and Glazer [17] hypothesized that a build-up of Bvd downregulated the biosynthesis of phycocyanobilin. Since that time, the genomes of many more cyanobacteria have been sequenced, and *bvdR* is present in most but not all cyanobacteria, including *Synechococcus* sp. PCC 7002 (hereafter *Synechococcus* 7002). In this study, we show that a *Synechococcus* 7002 *bvdR* mutant has lower levels of chlorophyll (Chl) *a*, mainly due to lower levels of photosystem I (PSI), and total phycobiliproteins (PBP), and that it accumulates more ROS than WT cells during exposure to high-light conditions. We suggest that bilirubin produced from Bvd by BvdR is the key component of a redox cycle that protects cyanobacterial cells from ROS.

## 2. Materials and Methods

*Strains and Growth Conditions.* WT and mutant strains of *Synechococcus* 7002 were grown in medium A supplemented with 1 mg NaNO_3_ mL^−1^ (denoted medium A^+^) [20]. To increase growth rates, the mutant strains were adapted to and grown in A^+^ medium with 10 mM glycerol added as a supplemental carbon source. The growth medium for mutant strains was additionally supplemented with 50 µg spectinomycin mL^−1^. Liquid cultures were slowly sparged with 1% (*v*/*v*) CO_2_ in air. Warm white fluorescent tubes provided continuous illumination at ~250 µmol photons m^−2^ s^−1^ for standard growth conditions. The adjustment of the growth light intensity was achieved through the addition of extra light sources or by shielding the light with a neutral density filter. The growth of the WT and mutant strains was monitored turbidometrically at 730 nm with a GENESYS 10 spectrophotometer (ThermoFisher Scientific, Waltham, MA, USA). *Escherichia coli* strain BL21 DE3 was used for overproduction of recombinant BvdR. 

*Phylogenetic and protein analysis.* Searches for protein homologs in databases were carried out with the BlastP program (www.ncbi.nlm.nih.gov/BLAST/ (URL accessed on 23 August 2023)) in GenBank, in the Integrated Microbial Genomes of the DOE Joint Genome Institute (https://img.jgi.doe.gov/ (URL accessed on 5 July 2023)) and in Cyanorak, the database for picocyanobacterial genomes (https://blast.sb-roscoff.fr/picocyanobacteria_all/ (URL accessed on 5 July 2023) [21]. Analyses of DNA and protein sequences were conducted using MacVector (MacVector, Inc., Apex, NC, USA). The crystal structure of *Synechocystis* 6803 BvdR with two molecules of Bvd and NADP was obtained from the protein data base (pdb: 5B3V) [19] and viewed with PyMol (https://pymol.org/2/ (URL accessed on 9 September 2023). Alphafold-2-generated [22] structure from *Synechococcus* 7002 BvdR sequence was then obtained (uniprot: B1XJL6).

From a curated dataset of BvdR protein sequences representing 11 taxonomic orders across 89 species of cyanobacteria (Table S1), maximum likelihood (ML) trees were inferred with rapid bootstrapping and subsequent ML search in RAxML v8.2.11 [23] executed through Geneious Prime v2023.2.1 (www.geneious.com), with trees rooted to *Gloeobacter violaceus* PCC 7421. Bootstrap support values were drawn on the best-scoring ML tree from 100 bootstrap replicates using the GAMMA model of rate heterogeneity and BLOSUM62 substitution matrix. The resulting tree was visualized in Geneious Prime v2023.2.1.

*Creation of a* bvdR *mutant.* Two segments of the *bvdR* gene (SYNPCC7002_A2324) were amplified by a polymerase chain reaction (PCR) using *Synechococcus* 7002 chromosomal DNA as template and Taq DNA polymerase. A 557-bp fragment including a portion upstream of the *bvdR* gene was amplified using 7002bvdR.up.F (5′ CGGTGCCTGAAATCAGTTTCCC 3′) and 7002BvdR.up.R (5′ ACGTGTTTCCCCGCTTCGAG 3′). This PCR product was digested with HincII (restriction site present within the *Synechococcus* 7002 *bvdR* gene) and was purified by electrophoresis on an agarose gel. A 702-bp fragment containing the 3′ portion of the *Synechococcus* 7002 *bvdR* gene was amplified using 7002bvdR.Dn.F (5′ TCGAAGCGGGGAAACACGTAG 3′) and 7002bvdR.Dn.R (5′ AAAAGCTTAGGTTTGGCC CAGGGCAGCCCG 3′) primers; this product was digested with BamHI (restriction site present within *bvdR* gene) and was purified using agarose gel electrophoresis. Plasmid pSRA81 containing the *aadA* gene, which confers resistance to spectinomycin, was digested with HincII and BamHI; the 1050-bp DNA fragment encoding *aadA* was also purified using agarose gel electrophoresis. The 557-bp *bvdR* upstream HincII fragment, the 1050 bp *aadA* HincII-BamHI fragment, and the 702-bp *bvdR* downstream BamHI-digested fragment were mixed and ligated together. This ligation mixture was then transformed into WT *Synechococcus* 7002 cells as described previously [24,25]. Transformants were selected on plates containing medium A^+^ supplemented with spectinomycin (50 µg/mL) and glycerol (10 mM) and solidified with 0.8% (*w*/*v*) Bacto-agar (Difco, ThermoFisher Scientific, Waltham, MA). Colonies were serially streaked on plates containing spectinomycin and then grown with and without glycerol in liquid medium. The full segregation of the WT and mutant alleles of *bvdR* was verified by PCR amplification using primers 7002bvdR.up.F and 7002bvdR.Dn.R. Template DNA from the WT strain produced a 1245-bp amplicon, while template DNA from the *bvdR* mutant produced a 2265-bp product.

*Production of Recombinant BvdR*. The *bvdR* gene from *Synechococcus* 7002 was amplified from genomic DNA using the primers 7002 bvdR.5.EcoRI.F (5′ AAGAATTCCATGGCAAAATTTCGAGTGGGCATTG 3′) and 7002 bvdR.HindIII.R (5′ AAAAGCTTAGGTTTGGCCCAGGGCAGCCCG 3′). The 1008-bp amplicon was digested with restriction enzymes EcoRI and HindIII and ligated with pETDUET-1 digested with the same enzymes. The cloned gene was verified using DNA sequencing. This bvdR-pETDUET-1 plasmid was transformed into BL21 DE3 cells, and colonies resistant to 100 μg ampicillin mL^−1^ were selected. A 25 mL overnight culture from one of these transformants was grown at 37 °C in Luria–Bertani (lysis broth) medium with shaking. This culture was used to inoculate a 1 L flask of autoinduction medium without lactose [26] until the culture reached 0.6 OD_600_. Isopropyl β-d-1-thiogalactopyranoside (IPTG) was added, and cells were allowed to grow at 18 °C with shaking for 18–20 h before they were harvested through centrifugation as described [27]. A lower growth temperature was used for induction of *bvdR* because we noticed that higher temperatures (30 °C) resulted in most of the protein being present in inclusion bodies within the *E. coli* cells. We also found that co-expression with heme oxygenase (*ho1*) from *Synechocystis* 6803 in pACYC-DUET (chloramphenicol was added to 34 μg mL^−1^, as described in reference [28]) resulted in higher soluble protein levels. Cell pellets were thawed, resuspended in ~10–250 mM NaPO_4_, 300 mM NaCl, 10 mM imidazole, pH 8.0, and supplemented with mini protease inhibitor cocktail (ThermoFisher Scientific, Waltham, MA, USA) and 0.01 mg ml^−1^ lysozyme (ThermoFisher Scientific, Waltham, MA, USA) prior to three passages through a French pressure cell at 138 MPa. The extract was centrifuged at 13,000× *g* for 15 min to remove any unbroken cells or inclusion bodies. The [His]_6_-tagged (HT) form of BvdR was purified using immobilized metal affinity chromatography over a cobalt affinity column (ThermoFisher Scientific, Waltham, MA, USA) following the protocol as described (The QIAexpressionist ™, fifth edition). The protein was eluted from the cobalt affinity column and dialyzed against 0.1 M K-PO_4_, pH 7.4, and 10% (*v*/*v*) glycerol and stored at −20 °C. Proteins were analyzed usnig SDS-polyacrylamide gels (15% *w*/*v*) and immunoblot analyses as described in reference [27] using rabbit antibodies (1:5000 dilution) generated against the recombinant BvdR of *Synechocystis* 6803 [17]. 

*BvdR Assays.* Stock enzyme solutions were prepared in 0.1 M K-PO4 buffer, at pH 7.4 and containing 10% (*v*/*v*) glycerol. The addition of the enzyme raised the pH of the assay mixture (see below) to 5.8. The optimal reaction conditions were as follows: 10% glycerol, 0.2 mg mL^−1^ bovine serum albumin, 0.1 M citrate buffer, pH 5.2 (made with Na-citrate and citric acid; the final pH of the reaction mixture after enzyme and Bvd addition was 5.8), 12 μg of BvdR, 100 μM NADPH, and 20 μM Bvd. The total volume was 1 mL, and the reaction was initiated by the addition of NADPH. Bvd (purchased from Frontier Scientific, Newark, DE, USA) or bilirubin (purchased from Gold Biotechnology St. Louis, MO, USA) were dissolved in a small amount of 0.1 N NaOH, and diluted into 0.1 M K-PO4, pH 7.4. The concentrations of Bvd and bilirubin were then determined as described in reference [17].

*HPLC Analyses of bilins.* The analysis of products formed upon the reduction in Bvd catalyzed by recombinant *Synechococcus* 7002 HT-BvdR was performed by reversed-phase high-performance liquid chromatography (HPLC) on a C_18_ column (3.9 μm × 150 mm) on a Waters E2695 HPLC equipped with a Waters 2996 photodiode array detector (Waters Corporation, Milford, MA, USA). A 1 mL reaction mixture containing 10 μg of HT-BvdR, 40 μM Bvd, 0.1 M citrate buffer, pH 5.2 (final pH 5.8), 10% (*v*/*v*) glycerol, 0.2 mg mL^−1^ bovine serum albumin, and 100 μM NADPH was incubated at room temperature for 30 min at 37 °C. This reaction mixture was then immediately diluted with 5 mL of 0.1% trifluoroacetic acid prior to loading on a C_18_ cartridge (Waters Corporation, Milford, MA, USA). For controls, 20 μM Bvd or bilirubin in 0.1 M of citrate buffer, pH 5.8, was diluted 5-fold with 0.1% trifluoroacetic acid (TFA) and treated in the same manner as the BvdR mixture. The C_18_ cartridge was washed with 0.1% TFA and eluted with solvent B (acetone/ethanol/water/acetic acid, 50:38:11:1 (*v*/*v*)). The eluate (2 mL) was dried in a vacuum centrifuge until the volume was ~250 μL; 250 μL of 0.1% TFA was then added, and the mixture was subjected to centrifugation for 5 min at 12,000× *g* prior to injection onto an analytical C_18_ column. The solvent systems used were water (solvent A) and solvent B. The mixture was loaded at a concentration of 50% solvent B and 50% water for 5 min at 0.75 mL min^−1^. A linear gradient was then applied from 50 to 100% solvent B over 45 min followed by 100% solvent B for 5 min. 

*Amplification of* cpcA *and* cpcB. Primers cpcB.1 (described in reference [29] and cpcB.2 (5′ CCTAGATATGTAAGCTTTAAGCTGGAT 3′) were used to amplify *cpcB* and primers cpcA.5 (5′ GGAGTTACCAGACATATGAAAACCCC 3′) and cpcA.3 (described in reference [29] were used to amplify *cpcA* from various chromosomal DNA templates purified from *Synechocystis* 6803 WT and *bvdR* mutants [17]. The PCR conditions used were [94 °C 30 s; 50 °C for 1 min; 72 °C for 1 min] × 35 cycles. PCR products were analyzed using electrophoresis on 0.8% (*w*/*v*) agarose gels prepared with Tris-acetate-EDTA buffer (0.4 M Tris-acetate (pH approximately 8.3); 10 mM Ethylenediaminetetraacetic acid (EDTA). After electrophoresis, gels were stained with ethidium bromide, and DNA fragments were visualized by fluorescence under UV light.

*Quantitation of Chl*, *PBP*, *and Carotenoids.* Spectroscopic measurements were performed on a GENESYS 10 spectrophotometer (ThermoFisher Scientific, Waltham, MA, USA). Pigments were extracted from *Synechococcus* 7002 cells with 100% methanol to determine the Chl content of cells [30,31]. The Chl *a* contents were compared on the basis of equivalent cell numbers as determined by equal OD_730_ values as previously described [32].

For measuring PBP content, *Synechococcus* 7002 cells in late-exponential growth phase (OD_730_ = 1.5–2.0) were collected and adjusted to 0.5 OD_730_ mL^−1^. The reduction in the PBP absorbance at 635 nm of liquid culture samples that had been heated at 65 °C for 5 min was used to calculate the PBP contents of samples as described [33,34]. 

To determine carotenoid content, the OD_730_ of cells was measured, and cells equivalent to 2.67 OD_730_ were pelleted through centrifugation at 12,000× *g* for 2 min. The following procedures were performed under very low light or in the dark. A 300 µL aliquot of acetone: methanol (1:2, *v*/*v*) was added to the cell pellets, and the mixtures were sonicated with 10 pulses. After centrifugation at 12,000× *g* for 2 min, the supernatants were removed and filtered through a 0.2 μm polytetrafluoroethylene syringe filter (6783-0402; Whatman, Clifton, NJ, USA). 

An aliquot (100 μL) was analyzed by reversed-phase HPLC. The sample was injected onto a C_18_ column using a protocol described for the analysis of all carotenoids in *Synechococcus* 7002 [25,35] with the absorbance of the eluate monitored at 491 nm. For each injected sample, the total areas under all peaks were added together. The total area for each sample was divided by the total area for the sample isolated from WT cells and the percentage of WT level was then calculated.

*Measuring ROS:* The cell-permeant reagent, 5-(and 6) chloromethyl-2′,7′-dichlorodihydroflourescein diacetate, acetyl ester (CM-H_2_DCFDA) (Invitrogen, Waltham, MA, USA), which is a derivative of reduced fluorescein, was used to detect ROS. This dye is non-fluorescent until acetate groups are removed by intracellular esterases and oxidation by ROS occurs within cells. Cells were grown to mid-exponential phase at normal light intensity (250 μE m^−2^ s^−1^), and their OD_730_ measured. Cells were harvested and resuspended in fresh A^+^ medium. For comparison, 5 mL of liquid culture of each strain was prepared and adjusted to OD_730_ = 0.4. A stock solution (5.0 mL) of each strain was prepared with OD_730_ = 0.4. Dimethylformamide was added to a fresh tube containing CM-H_2_DCFDA to produce a stock solution with 0.5 mM concentration. An aliquot (10 µL) of the reagent was added to 5.0 mL of cells to produce a final concentration of 1 μM. An aliquot (1.0 mL) of cells was then removed immediately and kept at reduced light intensity (~25 µmol photons m^−2^ s^−1^) on the lab bench (control). The remaining cells (4.0 mL) were placed at 2240 μE m^−2^ s^−1^ light intensity with sparging for 30 min. The cells were removed and placed in the dark for 15 min. The fluorescence emission at 520 nm (excitation at 495 nm) of 1.0 mL aliquots of each cell type were recorded in triplicate. The fluorescence emission of the control cells (treated with the CM-H_2_DCFDA but kept at reduced light intensity with no sparging) was also recorded. This control emission was subtracted from the fluorescence emission of the high-light treated cells. Finally, the OD_730_ for each of these strains was recorded after the incubations, and the fluorescence per 1.0 OD_730_ was calculated. The average fluorescence emission per OD_730_ was plotted along with the standard deviation for each cell type. 

## 3. Results

*Analysis of the* Synechocystis *6803* bvdR *mutant background.* One of us (W.M.S.) was involved in the initial characterization of BvdR in the cyanobacterium *Synechocystis* 6803 [17]. It was not possible to investigate the function of BvdR in *Synechocystis* 6803 further because the original *bvdR* mutant was lost in a freezer-thaw incident after Hurricane Katrina in 2005. However, when we attempted to remake the *bvdR* mutant by transforming *Synechocystis* 6803 again with DNA from the original plasmid construct used for the inactivation of *bvdR*, the mutant and WT alleles failed to segregate fully. The original *bvdR* mutant had undetectable levels of phycocyanin, so we amplified the *cpcB* and *cpcA* genes from the original *bvdR* mutant by PCR using the chromosomal DNA isolated from inviable cells of the original mutant, that has been stored at –80 °C, to determine if a secondary mutation might have occurred. The PCR product produced from the *cpcB* gene was smaller than the amplicon produced using template DNA from WT *Synechocystis* 6803 (see Figure S1). DNA sequencing revealed that a deletion of 122 bp had occurred within the *cpcB* gene (nucleotides 165–287), which resulted in a 41 amino acid deletion (55-96) within the coding region, including Cys-82. This explains why the original *bvdR* mutant strain was devoid of phycocyanin. This secondary mutation within *cpcB* may have permitted the segregation of the mutant and WT alleles of *bvdR* in *Synechocystis* 6803. This finding prompted us to renew studies to investigate the role of BvdR in cyanobacteria. 

*Phylogenetic relationships of cyanobacteria BvdR.* A survey of the sequenced genomes of cyanobacteria showed that most cyanobacteria, including the early diverging cyanobacterium, *Gloeobacter violaceus* PCC 7421 [36], contain the *bvdR* gene. *Parasynechococcus* (formerly called marine *Synechococcus*), *Prochlorococcus*, and *Acaryochloris* species all lack the *bvdR* gene. It seems that there have been lineage-specific losses of *bvdR* by these and other species, given that *Gloeobacter* and related strains have the gene. Interestingly, although they live in very similar mat communities and occupy similar ecological niches, *Thermosynechococcus* strains do not contain *bvdR*, but *Thermostichus* strains (formerly called *Synechococcus* sp.) [37] found in Yellowstone National Park hot springs or the Rupite hot springs [38] (*T. vulcaus*) contain one or even two copies of *bvdR*.

**Figure 1:**
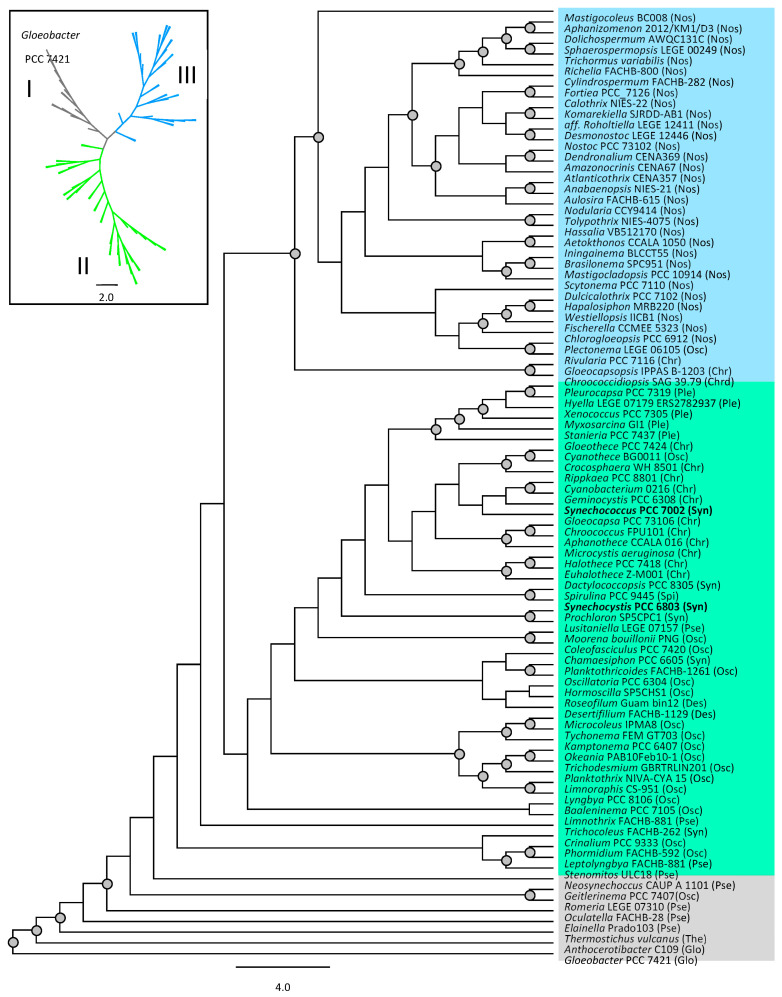
Maximum likelihood phylogenetic inference of cyanobacteria BvdR protein sequences. Evolutionary analysis was conducted in RAxML v 8.2.11 using the GAMMA model of rate heterogeneity and BLOSUM62 substitution matrix. The most likely tree inferred from 100 bootstrap replicates is shown with bootstrap values >50% identified as grey filled circles at the corresponding nodes. The basal cyanobacterium *Gloeobacter violaceus* PCC 7421 was used to root the tree. Taxa are labeled by genus and strain when available, otherwise by genus and species, followed by abbreviated taxonomic order in parentheses. Taxonomic orders include: Gloeobacterales (Glo), Thermostichales (The), Pseudanabaenales (Pse), Oscillatoriales (Osc), Synechococcales (Syn), Desertifilales (Des), Spirulinales (Spi), Chroococcales (Chr), Pleurocapsales (Ple), Chroococcidiopsidales (Chrd), and Nostocales (Nos). Three major clades were identified based on the unrooted phylogram (top left inset): I (grey—*Gloeobacter* PCC 7421 through *Neosynechococcus* CAUP A 1101); II (lime—*Stenomitos* ULC18 through *Pleurocapsa* PCC 7319); and III (blue—*Chroococcidiopsis* SAG 39.79 through *Mastigocoleus* BC008). See Table S1 for detailed information and metrics of all accessions used. The *Synechocystis* 6803 and *Synechococcus* 7002 sequences labels are in bold for easier viewing.

A phylogenetic analysis of BvdR from 89 species representing 11 orders resolved three main clades (Figure 1). For clades I and III, the tree topology followed a similar pattern as seen in the 16S rRNA tree of Strunecky et al. [39] and the RefSeq tree of Hirose et al. [40], with comparable placement of the most basal (Gloeobacterales, Thermostichales, and Pseudanabaenales) and more derived (Chroococcidiopsidales and Nostocales) species of represented orders to that of the current tree (Figure 1). Clade II was less clear in the recovery of distinguishable groupings of taxonomic orders, with 7 of the 11 orders represented in this clade (Figure 1). However, all members of Pleurocapsales and most members of Oscillatoriales and Chroococcales were generally clustered within their respective orders (Figure 1). Bootstrap support was moderate to high for clades I and III (>50%), but low for clade II, which may explain the disparate placement of some taxa (Figure 1). 

*Comparison of BvdR proteins:* The *Synechocystis* 6803 BvdR is 66.8% similar to BvdR from *Synechococcus* 7002 (7002 BvdR; Figure S2A). The structure of *Synechocystis* 6803 BvdR with two molecules of Bvd and one NADP^+^ [19] is shown in Figure S2B and is very similar to the predicted structure of a *Synechococcus* 7002 BvdR model obtained from Alpha-Fold2 (Figure S2C) [22], suggesting these two enzymes bind the same substrates and perform the same reaction.

**Figure 2:**
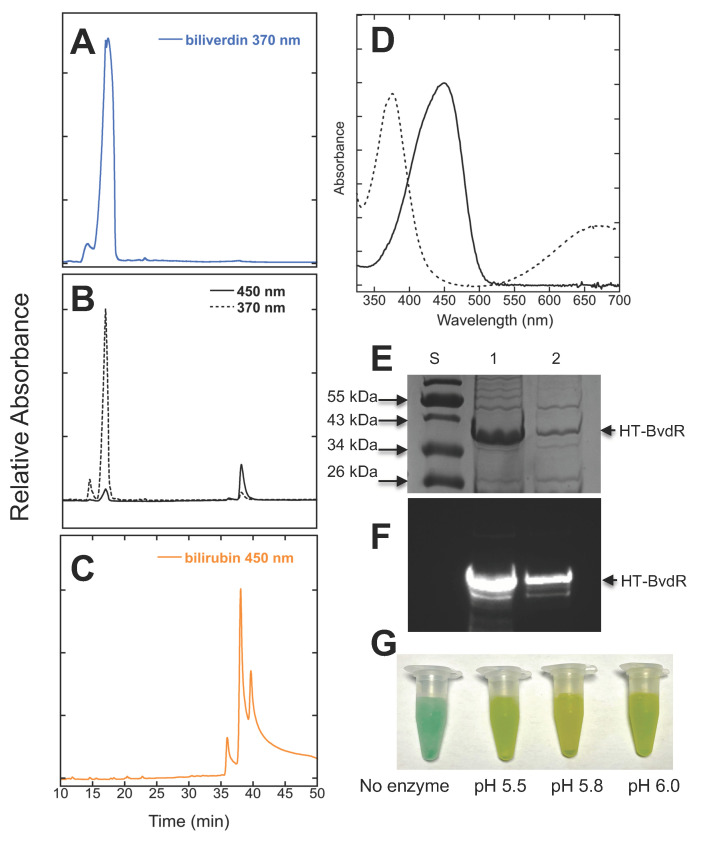
Analyses of biliverdin reductase. Bilins were separated using reversed-phase HPLC on a C_18_ column in panels A-C. Biliverdin was monitored at 370 nm and bilirubin was monitored at 450 nm. Elution profiles of biliverdin control (**A**), products of biliverdin reduction by NADPH catalyzed by *Synechococcus* 7002 BvdR after 30 min (**B**) and a bilirubin control (**C**) are shown. (**D**) The spectrum of biliverdin from the BvdR assay shown in panel B at 17 min (grey dotted line) and for the bilirubin product at 38.1 min (black solid line) are shown. (**E**) The purified 7002 HT-BvdR enzyme expressed with (lane 1) and without (lane 2) heme oxygenase was separated by SDS-PAGE followed by Coomassie blue staining. These proteins were purified from 1 L cultures grown in the same way, and the proteins were concentrated to 1 mL final volume after dialysis. The same volume (6 μL) was loaded for both, illustrating the higher yield of soluble protein when heme oxygenase (which produces biliverdin) was present in cells. (**F**) Immunoblots of the purified *Synechococcus* 7002 HT-BvdR samples, which were probed with antibodies raised against the *Synechocystis* 6803 BvdR enzyme, are shown. The antibodies cross-reacted with a single major polypeptide at ~37 kDa. (**G**) Picture of 1 mL assays after 30 min, containing no enzyme (final pH 5.8) or containing 7002 BvdR at various final pH values. See Materials and Methods section for details.

*Overexpression*, *purification, and assays of* Synechococcus 7002 *bvdR*. The *Synechococcus* 7002 *bvdR* gene was cloned into a pET-DUET expression vector and recombinant BvdR was produced in *E. coli* as a [His]_6_-tagged (HT) protein, denoted HT-BvdR. The recombinant HT-BvdR was purified using immobilized metal affinity chromatography. A reaction mixture containing Bvd, NADPH, bovine serum albumin, glycerol, citrate buffer, pH 5.8, and HT-BvdR was incubated for 30 min at 37 °C as described in the Materials and Methods section. The Bvd and bilirubin control mixtures (no HT-BvdR) in 1 mL mock reactions were treated similarly prior to analysis on a C_18_ reversed-phase column. The elution profiles were monitored at 370, 680, and 450 nm (Figure 2). Bvd has a retention time of 17 min and was detected at 370 nm (Figure 2A). The elution profile of the BvdR reaction mixture is shown in Figure 2B. The retention time of the Bvd is identical to that shown in panel A. The second observed peak in the reaction mixture in Figure 2B with a retention time of 38.1 min and an absorbance peak of 450 nm matches that of the bilirubin control (Figure 2C). The spectra of the Bvd substrate with a retention time of 17 min in Figure 2B, and of the bilirubin product produced by BvdR at 38.1 min (Figure 2B), are shown in Figure 2D. The bilirubin product formed in the reaction with BvdR (Figure 2B) has the characteristic absorbance peak at 450 nm (see Figure 2D). 

The BvdR from *Synechococcus* 7002 was not very soluble when produced at 30 °C in *E. coli*, and the protein had to be produced at 18 °C in order to purify enough protein to perform the assays. When we co-expressed this protein with heme oxygenase from *Synechocystis* 6803 (in the hope that the presence of Bvd would result in a more soluble enzyme), we had a higher yield of the enzyme (Figure 2E); however, the Bvd present in these cells did not form stable complexes with BvdR because, after elution from the Co-affinity column, there was no Bvd bound as judged using absorption spectroscopy. When the proteins separated by SDS-PAGE were probed with antibodies made against the recombinant BvdR of *Synechocystis* 6803, one major protein band was recognized, corresponding to the predicted size of the HT-BvdR at 36.9 kDa (Figure 2F). The optimal pH of the *Synechococcus* 7002 BvdR enzyme was 5.8, the same as reported for the *Synechocystis* 6803 BvdR enzyme [17] as determined by viewing the yellow color of 1 mL assays after 30 min when the final pH of the assays was varied from pH 5.5 to 6.0 (Figure 2G). Together, these results demonstrate that *Synechococcus* 7002 BvdR can reduce Bvd to bilirubin using NADPH as the coenzyme. 

**Figure 3:**
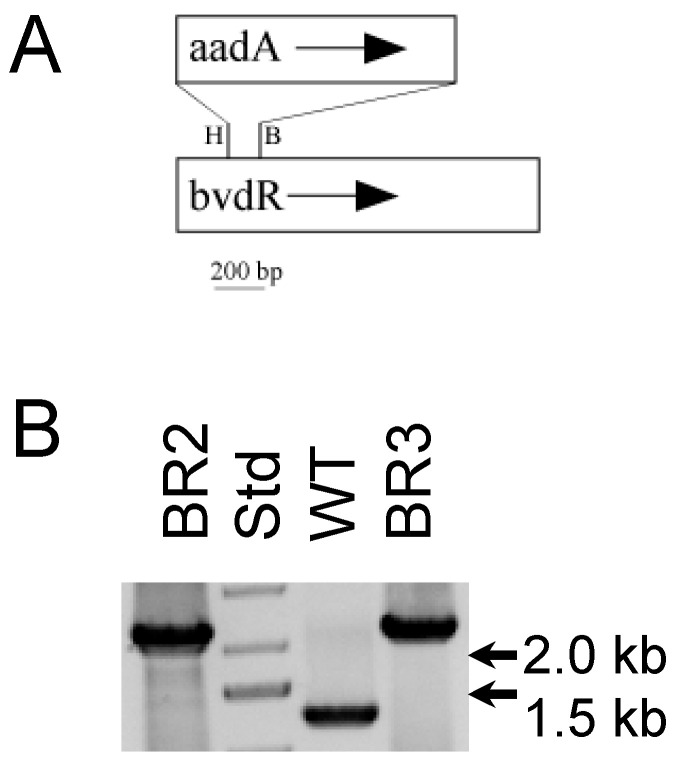
Physical map of the biliverdin reductase mutant construct and PCR of *bvdR* alleles. (**A**) Physical map of the *bvdR* gene from *Synechococcus* 7002 showing how the *aadA* gene, conferring resistance to spectinomycin, was inserted. Restriction enzyme sites indicated are H = HincII and B = BamHI. (**B**) Agarose gel electrophoresis of *bvdR* alleles amplified (using 7002bvdR.up.F and 7002bvdR.Dn.R primers) from the chromosomal DNA isolated from the BR2 mutant, BR3 mutant, and WT cells. Size standards are loaded in the lane marked “Std” with the sizes indicated at the right. The WT *bvdR* gene produced a 1245 bp product, while the mutant allele produced a product of 2265 bp.

*Construction of* Synechococcus *7002* bvdR *mutant.* We made a *bvdR* mutant in *Synechococcus* 7002 in the hope of discovering a function for BvdR in cyanobacteria, as described in the Materials and Methods section. The total DNA was isolated from cells from two independent transformants (denoted BR2 and BR3) and the WT; the *bvdR* PCR amplicons produced from these chromosomal DNAs were separated using agarose gel electrophoresis (Figure 3). The size of the products amplified from two *bvdR* isolates, designated BR2 and BR3, was 2265 bp, whereas the product amplified from the WT was 1245 bp as predicted; no amplicons with the size expected for the WT *bvdR* allele were detected from either of the *bvdR* mutant strains. 

*Phenotypic analysis of the* Synechococcus 7002 bvdR *mutant*. Because strains BR2 and BR3 appeared to be identical, all further studies were performed with strain BR2. The cells of strain BR2 were more yellow-green in color than the WT cells and whole-cell absorption spectra indicated that PBP levels were lower than in the WT cells (Figure S3A,B). The PBP, Chl, and carotenoid contents were measured for the WT cells and the BR2 mutant cells (Table 1; Figure S4). The PBP and Chl contents of the BR2 cells were only about one third of the WT levels, whereas carotenoid levels were ~50% higher in the mutant cells than in the WT cells. Although carotenoid levels increased, the relative proportions of the individual carotenoids were not substantially different in WT and the BR2 mutant strain (Figure S4).

**Figure 4:**
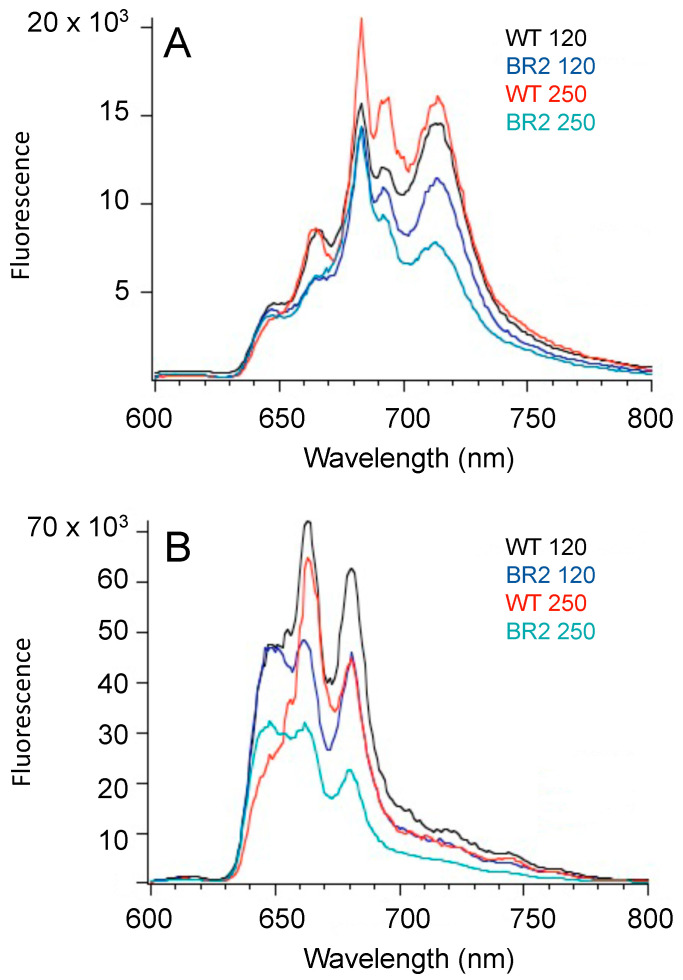
Low-temperature fluorescence emission spectra of the *Synechococcus* 7002 WT and BR2 mutant strain. (**A**) Low-temperature (77 K) fluorescence emission spectra of intact cells of the BR2 mutant and WT at 120 μE m^−2^ s^−1^ and 250 μE m^−2^ s^−1^. The excitation wavelength was 440 nm, which preferentially excites Chl *a*. (**B**) Low-temperature 77 K fluorescence emission spectra with excitation at 590 nm, which preferentially excites PBP. Cells were adjusted to the same cell density at 0.5 OD_730_ mL^−1^. Each spectrum is the average of four independent measurements.

The differences in the PBP and Chl levels within cells can also be revealed and compared using fluorescence emission spectroscopy at a low temperature (77 K). Fluorescence emission spectra at 77 K were measured with the preferential excitation of Chl *a* at 440 nm for intact cells of the WT and the BR2 mutant grown to the same cell density at normal light intensity (250 μE m^−2^ s^−1^) or at medium light intensity (120 μE m^−2^ s^−1^) (Figure 4A). For the WT cells, the 715 nm emission occurs from Chls associated with PSI complexes, the 695 nm emission from Chls associated with CP47 (PsbB) of PS II, and the 685 nm emission from Chls associated with CP43 (PsbC) of PSII [24]. Consistent with the lower pigment content of the cells of the BR2 mutant, the overall fluorescence emission from the Chl proteins (PSI and PSII) was lower in amplitude than for the WT cells. The emission from PSI in the BR2 mutant was specifically lower in cells grown at normal light intensity.

The excitation at 590 nm preferentially excites PBP in cyanobacterial cells [41]. The WT cells display three major fluorescence emission peaks (Figure 4B); the emission peak at 645 nm is mostly due to phycocyanin, the 665 nm emission peak is mostly due to allophycocyanin, and the 680 nm emission peak is mostly due to fluorescence emission from the terminal emitters of the PBS (ApcD and ApcE) as well as Chls associated with PSII [42]. Based upon a comparison of the fluorescence emission amplitudes from equal numbers of cells, the fluorescence amplitude was greatly reduced, especially from the 665 nm and the 680 nm emission peaks, in cells of the BR2 mutant (Figure 4B). Overall, the emission amplitude from all PBP was markedly reduced in the BR2 cells grown at normal or medium light intensity, which agrees with the overall reduction in PBP content in the BR2 cells (Table 1) as well as the yellowish-green color of the mutant cells (Figure 3A). 

PBS were isolated on sucrose gradients from the WT and BR2 cells. Based upon the behavior on sucrose gradients, the PBS in the mutant strains seemed to be similar in size and density to those in the WT (Figure S5A). Furthermore, the absorbance (Figure S5B) and fluorescence emission were virtually identical. These data indicate that the *bvdR* mutant has reduced levels of PBPs, but those PBPs that are produced are normal and are assembled into PBS in a manner similar to the WT. Therefore, it appears that the *bvdR* mutants are making fewer total PBPs rather than reducing the amount of a particular PBP. Consistent with having fewer total PBPs and PSI, the BR2 isolate grew more slowly than the WT at normal light intensity (250 μE m^−2^ s^−1^) (Figure S6).

**Figure 5:**
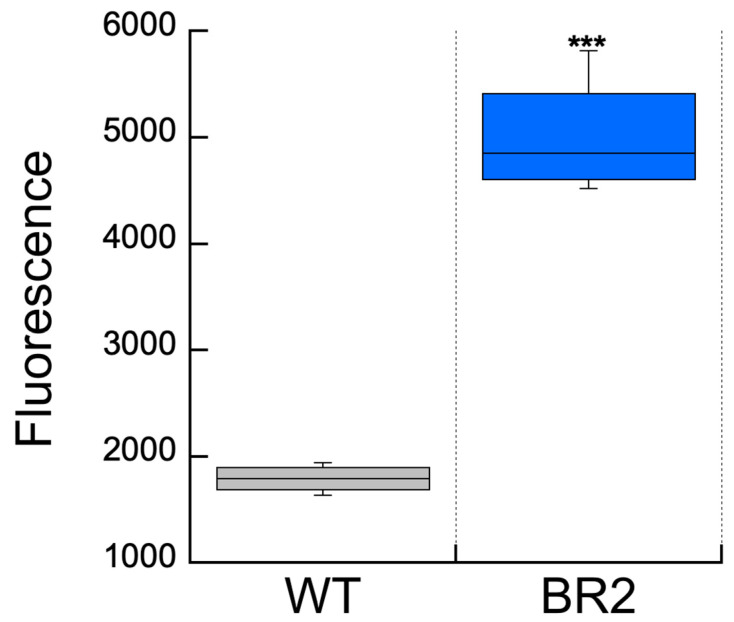
Measurement of ROS within WT and BR2 mutant cells. Cells were loaded with the fluorescein dye derivative CM-H_2_DCFDA, incubated in high light (2240 μE m^−2^ s^−1^), and the relative average fluorescence measured as described in the Materials and Methods. Box and whisker plots with error bars indicate the standard deviation for the three measurements for each cell type (WT cells (dark grey) and BR2 mutant cells (blue). The mean for each data set is indicated by the horizontal line. Asterisks indicate statistical significance levels of ROS for the BR2 mutant compared to WT using a two-tailed *t*-test, assuming unequal variance performed using MS excel: *** *p* < 0.001.

*Measurement of ROS* in vivo. The cell-permeant ROS indicator, CM-H_2_DCFDA, was used to determine if there was a higher level of ROS in the BR2 mutant compared to the WT cells. After the exposure of cells to 2240 μE m^−2^ s^−1^ for 30 min, followed by a 15 min incubation in the dark, the fluorescence emission at 520 nm was measured. The ROS levels detected using this method for these cells are presented in Figure 5. The ROS level in the BR2 mutant strain was significantly higher, 2.8-fold, than in the WT cells.

## 4. Discussion

The goal of this study was to probe the function of BvdR in cyanobacteria. We were unable to regenerate the *Synechocystis* 6803 *bvdR* knockout strain using the original plasmid constructs used to generate the previously reported mutant (Schluchter and Glazer, 1997). When the *cpcB* gene was amplified from the chromosomal DNA isolated from the bvdR3.2G mutant described in the original study [17] and sequenced, we found that the amplicon contained a 122-bp deletion within the coding region of the *cpcB* gene, explaining why the original *Synechocystis* 6803 mutant lacked phycocyanin. The absence of phycocyanin would reduce the overall photosynthetic rate in the strain several-fold [43,44], which presumably would result in lower ROS levels. Reducing the oxidative stress levels in cells would lessen the requirement for a functional BvdR, which thus might have allowed the segregation of the WT and mutant alleles of the *bvdR* gene after transformation. 

The *bvdR* mutant strain described here, called BR2, was generated in *Synechococcus* 7002, a cyanobacterial strain that can tolerate and grow at much higher light intensities than *Synechocystis* 6803 [45]. The BR2 cells showed lower levels of PBPs and Chl than the WT strain but higher levels of carotenoids (Table 1 and Figure S4). The BR2 cells also appeared to be making fewer total PBS overall (Figure S5). As a result of the lower content of photosynthetically active pigments, the BR2 mutant cells grew somewhat slower than the WT cells (Figure S6). 

The ROS levels were significantly higher in the BR2 compared to the WT cells after exposure to high light (Figure 5). This is consistent with the purported redox protection role of BvdR in mammals, where it plays an important role in redox protection [6]. In cultured human cells, the depletion of BvdR (BVRA isoform) by RNAi knockdowns leads to apoptosis and increased sensitivity to oxidative stress [6]. When a free radical initiator was added, bilirubin was degraded in these cells, generating Bvd [3,6]. These results suggested that mammalian BvdR is a part of an important catalytic cycle, regenerating bilirubin after it is oxidized by ROS in vivo. This allows an amplification so that the nM levels of bilirubin can catalytically overcome oxidants at concentrations that are up to four orders of magnitude higher [6]. 

**Figure 6:**
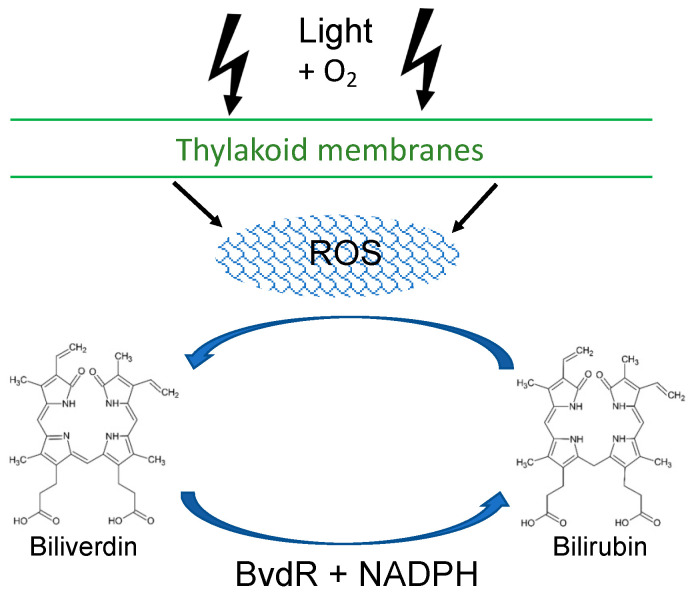
Model for the role of BvdR in protecting cyanobacteria from ROS. Oxygen generated from photosynthesis reacts with metals present in the electron transfer complexes and photosystems present in the thylakoid membranes, producing ROS, including singlet oxygen (^1^O_2_), superoxide anions (O_2_^−^), hydrogen peroxide (H_2_O_2_), and hydroxyl radicals (OH^•^). These can react with bilirubin to generate biliverdin. BvdR then uses NADPH (produced by Ferredoxin NADP^+^ reductase [57]) and re-reduces biliverdin to form bilirubin.

Cyanobacterial cells produce oxygen at high levels during photosynthesis through the actions of the photosynthetic electron transport complexes. ROS are generated by metals within the reaction centers, such as [4Fe–4S] clusters in PSI and ferredoxins, and Fe^2+^ in PSII, Mn^2+^ in the PSII oxygen evolving complex, Mg^2+^ in Chl, present in both PSI and PSII, and Cu^2+^ in plastocyanin [46,47,48], as well as metal centers within the NADH dehydrogenase and the cytochrome *b*_6_*f* complex during respiratory electron transport [49,50]. ROS, including singlet oxygen (^1^O_2_), the superoxide anion (O_2_^−^), hydrogen peroxide (H_2_O_2_), and the hydroxyl radical (OH^•^) [51,52], can oxidize the thiols of the cysteine residues in proteins, cause the photoinhibition of the D1 protein in PSII [53], damage the metal cofactors in many enzymes, damage DNA, and damage lipids by peroxidation [48,54]. To protect against ROS generation, cyanobacteria have enzymes including superoxide dismutase, catalases, peroxidases, peroxiredoxins, and rubrerythrin. Superoxide dismutase converts the superoxide anion to hydrogen peroxide, with peroxidases, catalases, peroxidredoxins, and rubrerythrins converting hydrogen peroxide to water [46,47,48]. In addition, carotenoids and smaller metabolites such as ascorbate, glutathione, and α-tocopherol also play important roles in the detoxification of ROS [46,55,56]. Given the increased ROS levels and lower pigment levels found in the BR2 mutant in *Synechococcus* 7002, and our discovery that the original *bvdR* mutant in *Synechocystis* 6803 contained a loss of *cpcB* that was likely required to facilitate the loss of *bvdR*,, we suggest that, in cyanobacteria, BvdR may play an important role in a catalytic cycle of regeneration of the lipid-soluble bilirubin after it reacts with ROS, producing Bvd (Figure 6). Cyanobacteria lacking BvdR must have other mechanisms to compensate for ROS.

It is not surprising that *Gloeobacter* spp., and other deeply branching groups of cyanobacteria, have BvdR; this enzyme may have been an important response to the selective pressure these organisms experienced when oxygenic photosynthesis first arose. Oxygen is required for heme oxygenase activity to produce Bvd [58]. Indeed, the timing of the evolution of antioxidant enzymes like superoxide dismutase in cyanobacteria suggests these enzymes were important contributors to the success of these organisms responding to the effects of newly created ROS [47]. Members of the *Vampirovibrionia*, which are thought to have given rise to the cyanobacteria [59], do not contain *bvdR*, suggesting this gene was an important early acquisition to facilitate the oxygenic lifestyle. It may be more surprising that some cyanobacterial lineages evidently lost *bvdR*. For example, *Thermosynechococcus* spp. do not have *bvdR*, while *Thermostichus* spp, which occupy a similar niche, have one or even two copies of *bvdR*. *Prochlorococcus* spp. do not contain heme oxygenase or *bvdR*; however, *Parasynechococcus* spp. (formerly the marine *Synechococcus*) contain PBP and heme oxygenase but presumably lost *bvdR* [60]. Nonetheless, it appears that the BvdR enzyme has a long evolutionary history, similar to superoxide dismutase enzymes [47]. Our phylogenetic analysis of cyanobacterial BvdR shows a similar pattern as was observed for superoxide dismutase enzymes [47]. The ancestor to chloroplasts was likely a N_2_-fixing, unicellular cyanobacterium of the order Chroococcales, a group that contains *bvdR* [61]; however, plant chloroplasts do not contain *bvdR* (see below) [62,63,64]. 

The members of Actinobacteria have a structurally and mechanistically distinct family of BvdR enzymes that rely on the deazaflavin cofactor F_420_H_2_ [65]. These enzymes have been characterized from *Mycobacterium tuberculosis* [65] and *Streptomyces atratus* [66], and both produce bilirubin from Bvd using electrons from the F420 cofactor rather than NADPH. These enzymes are thought to participate in the protection of these bacteria from ROS generated in *M. tuberculosis* infections of the lung [65] or from nitric oxides produced by *Streptomyces* spp. [66].

Plants contain heme oxygenase, and its product, Bvd, is the substrate for the HY2 enzyme that produces phytochromobilin [67], the chromophore bound to the plant photoreceptor phytochrome [68]. However, plants do not contain a gene for BvdR [62,63,64]. Very recently, Ishikawa et al. demonstrated that bilirubin is formed non-enzymatically in chloroplasts of multiple plant species when Bvd reacts with NADPH [69]. This group detected bilirubin using the bilirubin-dependent fluorescent eel protein UnaG [70] while imaging live plant cells [69]. Bilirubin was detected in the angiosperms *Nicotiana benthamiana* and *Arabidopsis thaliana* as well as in the early diverging plant, the liverwort *Marchantia polymorpha*, suggesting this pigment is likely present in all plants [69]. Bilirubin had been detected in plants previous to this study [71]. Importantly, Ishikawa et al. showed that the ectopic expression of rat BvdR in plant chloroplasts increased bilirubin levels, while decreasing ROS levels [69]. The amount of bilirubin produced in plants non-enzymatically is estimated to be much less than the amount produced by BvdR in mammals. Ishikawa et al. postulate that this level of bilirubin is sufficient and important for plants to contribute to scavenging ROS because plants do not have as much free heme that needs to be degraded as mammals do (due to the degradation of hemoglobin) [69]. Plants also have a much higher level of NADPH produced in chloroplasts as a result of photosynthetic electron transport, allowing it to react with Bvd to produce bilirubin. 

In plants, tetrapyrroles, including heme and bilins, regulate photosynthetic gene expression as retrograde signaling molecules from the plastid to the nucleus [72,73]. ROS produced during photosynthetic electron transport also act as retrograde signaling molecules [74,75]. It seems that bilirubin and/or the ROS species may regulate specific nuclear genes. Similarly, in mammals, bilirubin has been shown to behave like a hormone [76,77,78]. 

Oxygenic phototrophs regulate tetrapyrrole biosynthesis in response to light and oxygen both transcriptionally and post-transcriptionally [79,80,81,82,83,84,85,86,87]. The mechanism for the downregulation of the pigment production in the BR2 mutant remains to be determined. However, it appears that this compensatory downregulation of pigmented proteins would be an important mechanism for lowering ROS production in cells. This may explain why the *cpcB* deletion was likely required to allow the segregation of the *bvdR* mutant in *Synechocystis* 6803, a cyanobacterium with less tolerance to high light stress than *Synechococcus* 7002. We present here the first evidence that BvdR is important in reducing the damage due to ROS in cyanobacteria. BvdR was estimated to be present at 200–450 copies per cell in *Synechocystis* 6803, which is a fairly high level for an enzyme [88]. More work will need to be carried out to determine the importance of the BvdR enzyme in dealing with ROS. However, its wide distribution in cells that are known to suffer physiologically from ROS (Figure 1), which is commonly produced because of their oxygenic photosynthetic lifestyle, is consistent with an important role in detoxifying ROS.

## Figures and Tables

**Table 1:** Relative PBP, Chl, and Carotenoid Contents of Cells from *Synechococcus* 7002 WT (WT) and *bvdR* mutant strain BR2 (BR2).

Strain	Relative PBP Content (%) ^1^	μg Chl (OD_730_ · mL)^−1^	Relative Carotenoid Content (%)
WT	100	3.56 ± 0.28 (100%)	100
BR2	32.5	1.30 ± 0.40 (36.5%)	150

^1^ Pigment contents were determined three times with independent biological replicates. Representative results are presented for PBP and carotenoids.

## Data Availability

The data presented in this study are available in the Supplementary Material.

## Supplementary Materials

The following supporting information can be downloaded at: https://www.mdpi.com/article/10.3390/microorganisms11102593/s1, Table S1: BvdR sequences (excel file loaded separately); Figure S1: PCR analysis of the *cpcA* and *cpcB* genes from two *Synechocystis* 6803 biliverdin reductase (*bvdR*) mutant chromosomal DNAs; Figure S2: Biliverdin Reductase sequences alignment and structures.; Figure S3: Appearance and absorbance spectra of WT and mutant cells; Figure S4: HPLC analyses of carotenoids extracted from WT and BR2 cells; Figure S5: Analyses of phycobilisomes (PBS) from WT and the BR2 mutant; Figure S6: Growth curves of WT and BR2 mutant cells.
