# Supplementary material for: Loss of Biliverdin Reductase Increases Oxidative Stress in the Cyanobacterium Synechococcus sp. PCC 7002
Source: Microorganisms. 2023 Oct 20;11(10):2593. doi: 10.3390/microorganisms11102593 (PMC10608806; doi:10.3390/microorganisms11102593)

Supplementary Materials for

Loss of Biliverdin Reductase Increases Oxidative Stress in Cyanobacterium *Synechococcus* sp. PCC 7002

Wendy M. Schluchter [1,*], Courtney H. Babin [1], Xindi Liu [1], Amori Bieller [1], Gaozhong Shen [2], Richard M. Alvey [2,3] and Donald A. Bryant [2]

1 Department of Biological Sciences, University of New Orleans, New Orleans, LA 70148, USA; cchymel@my.uno.edu (C.H.B.); xliu9@my.uno.edu (X.L.); abiell3@lsu.edu (A.B.)

2 Department of Biochemistry and Molecular Biology, The Pennsylvania State University, University Park, PA 16802, USA; gxs22@psu.edu (G.S.); ralvey@iwu.edu (R.M.A.); dab14@psu.edu (D.A.B.)

3 Biology Department, Bloomington, Illinois Wesleyan University, Bloomington, IL 61702, USA

* Correspondence: wschluch@uno.edu

Table S1: BvdR sequences (excel file loaded separately)

Figure S1: PCR analysis of the *cpcA* and *cpcB* genes from two *Synechocystis* 6803 biliverdin reductase (*bvdR*) mutant chromosomal DNAs.

Figure S2: Biliverdin Reductase sequences alignment and structures.

Figure S3: Appearance and absorbance spectra of WT and mutant cells.

Figure S4: HPLC analyses of carotenoids extracted from WT and BR2 cells.

Figure S5: Analyses of phycobilisomes (PBS) from WT and the BR2 mutant.

Figure S6: Growth curves of WT and BR2 mutant cells.

Figure S1: PCR analysis of the *cpcA* and *cpcB* genes from two *Synechocystis* 6803 biliverdin reductase (*bvdR*) mutant chromosomal DNAs. Chromosomal DNA preparations stored in a –80 °C freezer were used as templates for PCR amplification of *cpcA* and *cpcB* genes in two *bvdR* mutant strains. These mutants were described and characterized in [19]. The expected sizes of the *Synechocystis* 6803 WT *cpcA* gene is 540 bp and of the WT *cpcB* gene is 600 bp. In [19], br3.2G was the transformant that segregated and produced no phycocyanin whereas transformant br3.2A did not segregate. The *cpcB* gene amplified from this strain is 122 bp smaller (as verified by DNA sequencing) than amplicons from the WT or br3.2A. The *cpcA* product sizes are the expected size in all cases.

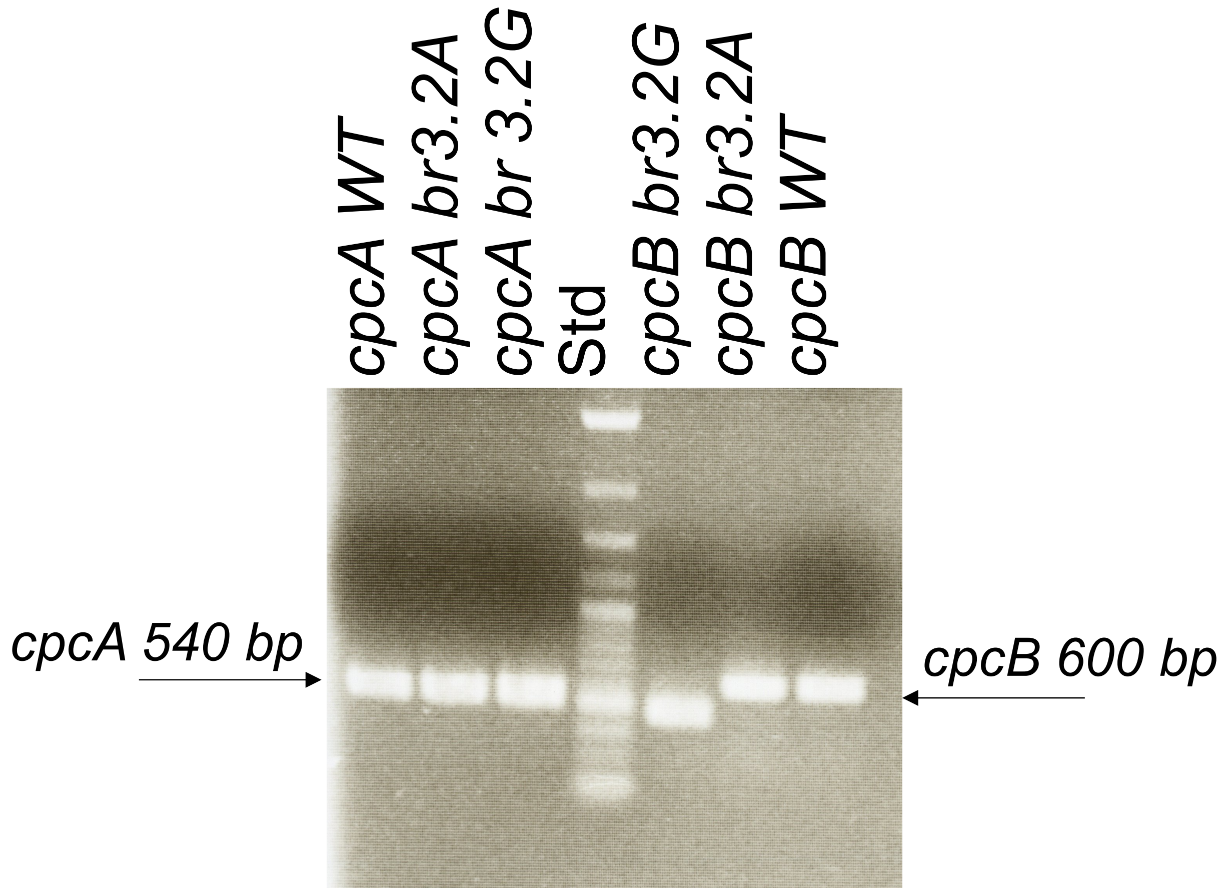

Figure S2: Biliverdin Reductase sequences alignment and structures. A. Alignment of BvdR protein sequences from *Synechococcus* 7002 and *Synechocystis* 6803 using ClustalW. Similar amino acid residues are denoted with light shading and identical matches are indicated with darker shading. B. Crystal structure of *Synechocystis* 6803 BvdR in brown (pdb: 5B3V) with two molecules of Bvd and one molecule of NADP+ in pink [18]. C. Alphafold-2 generated [22] model for *Synechococcus* 7002 BvdR (uniprot: B1XJL6).

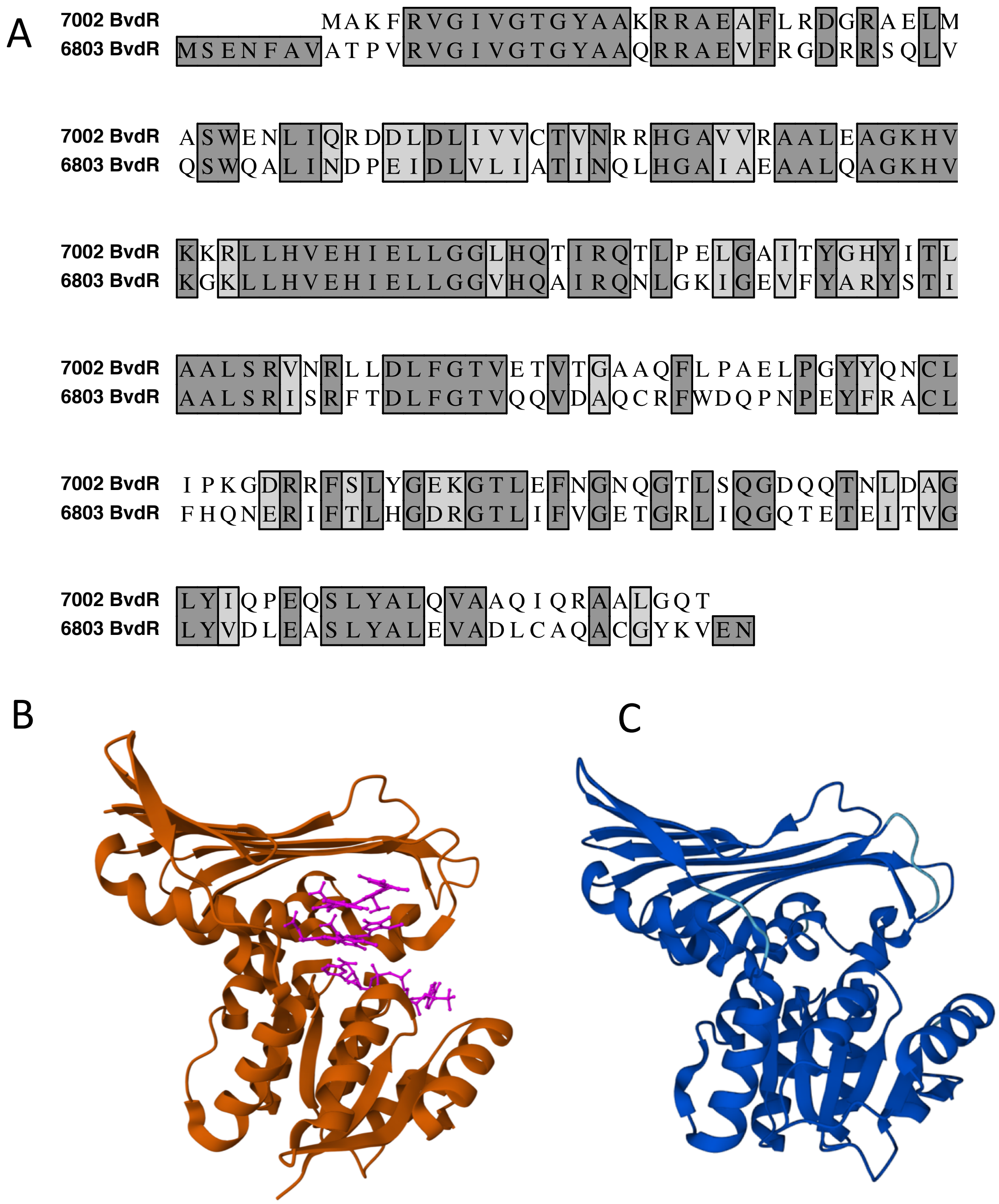

Figure S3: Appearance and absorbance spectra of WT and mutant cells. A. *Synechococcus* 7002 cultured cells after growth at 250 ◎E $\mathrm{m^{-2}\ s^{-1}}$. Cultures pictured are WT cells (left) and the BR2 strain (right). B. Whole-cell absorbance spectra of these two cultures taken in white light.

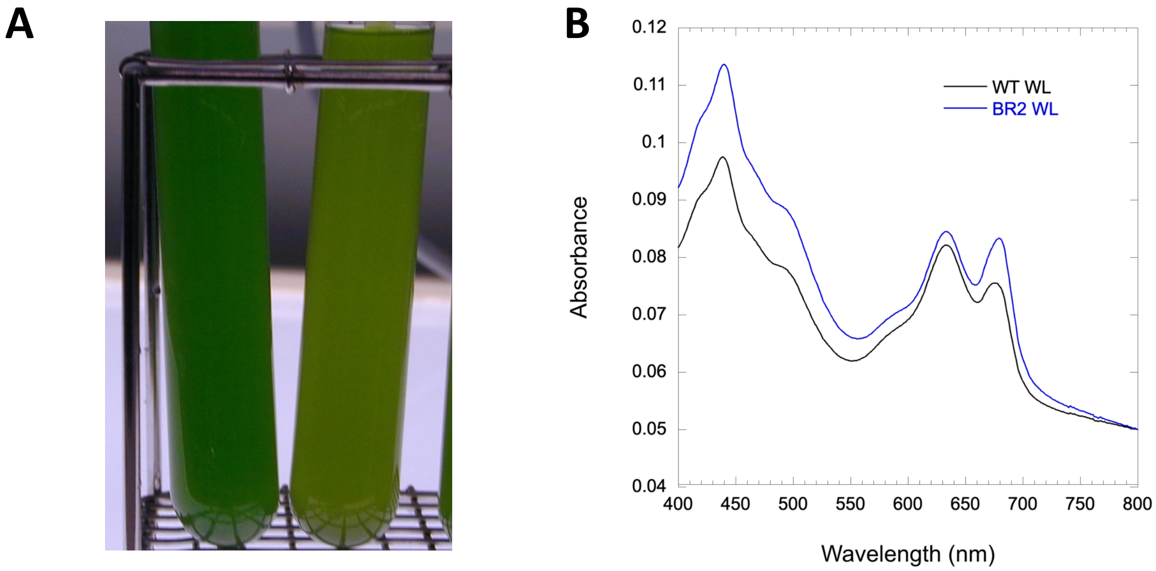

Figure S4: HPLC analyses of carotenoids extracted from WT and BR2 cells. Each sample was injected onto a C_{18} column with the absorbance of the eluate monitored at 491 nm [25,35]. Carotenoid identities are 1, synechoxanthin; 2, myxol-2′-fucoside; 3, zeaxanthin; 4 cryptoxanthin; 5, echinenone; 6, lycopene; 7, β-carotene.

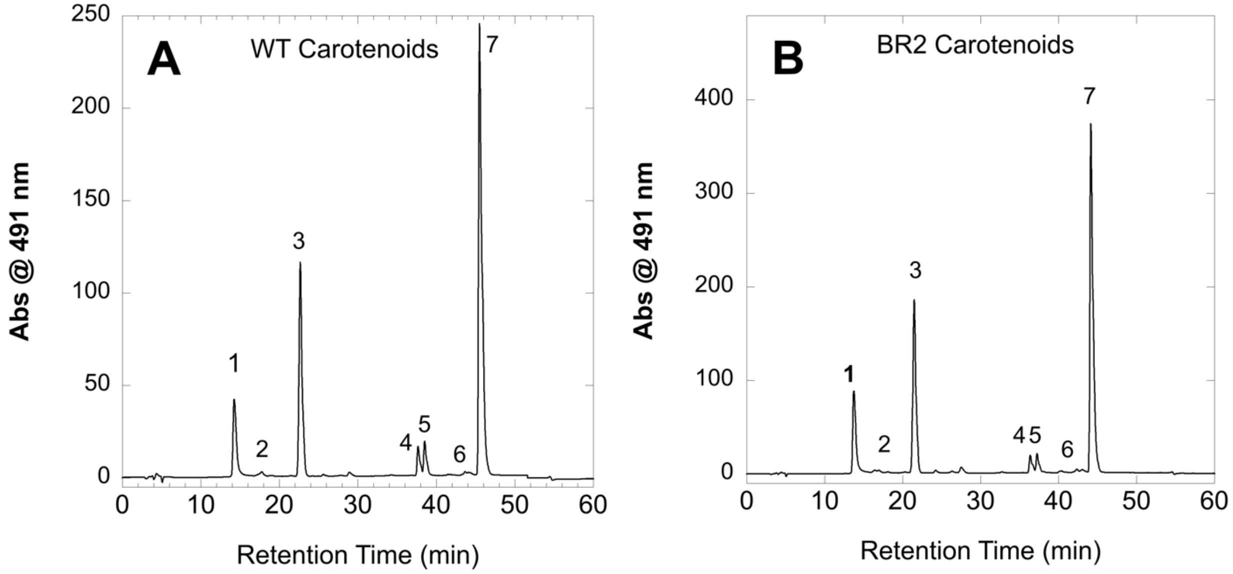

Figure S5: Analyses of phycobilisomes (PBS) from WT and the BR2 mutant. **A.** Sucrose density gradients are pictured for extracts from WT and BR2 mutant cells. **B.** PBS spectra were acquired as shown from the PBS bands indicated in A. Note that the PBS content of cells was much lower in the BR2 mutant strain, but that their composition is very similar except for increased contaminating Chl-containing membranes in the BR2 sample (shoulder at 680 nm).

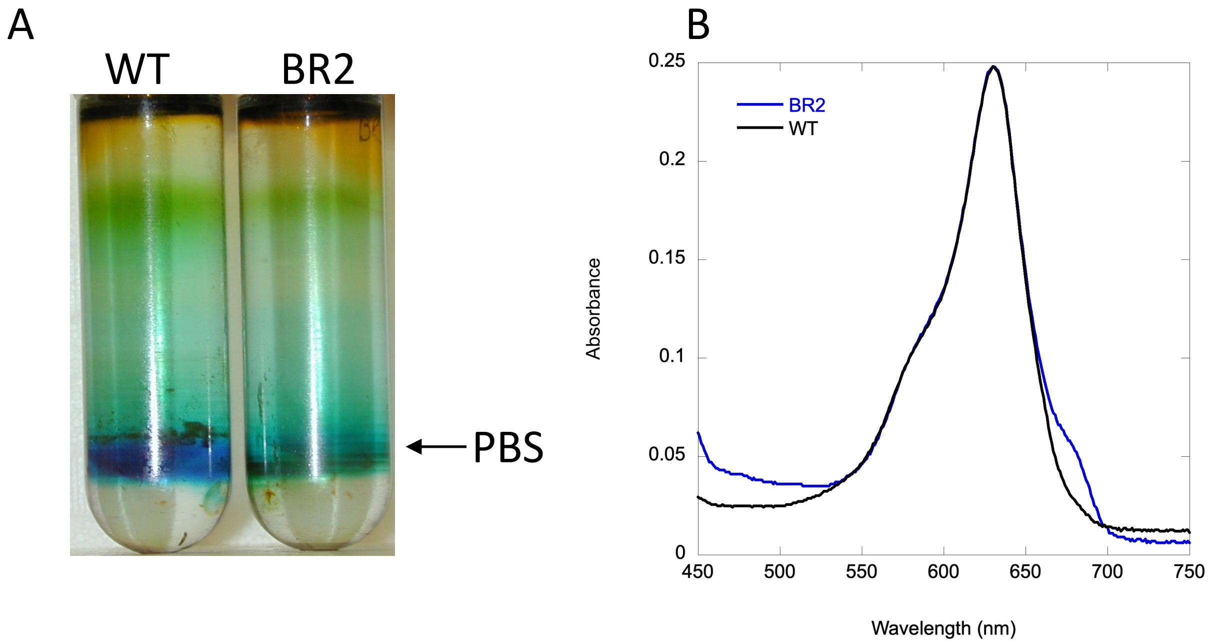

Figure S6: Growth curves of WT and BR2 mutant cells. Cell growth at 250 μE m^{-2} s^{-1} was monitored at OD_{730} over time. Error bars indicate standard deviations (SD) of three replicate experiments. For some data points, the error bars obtained using three replicates are smaller than the symbols and thus are not visible.

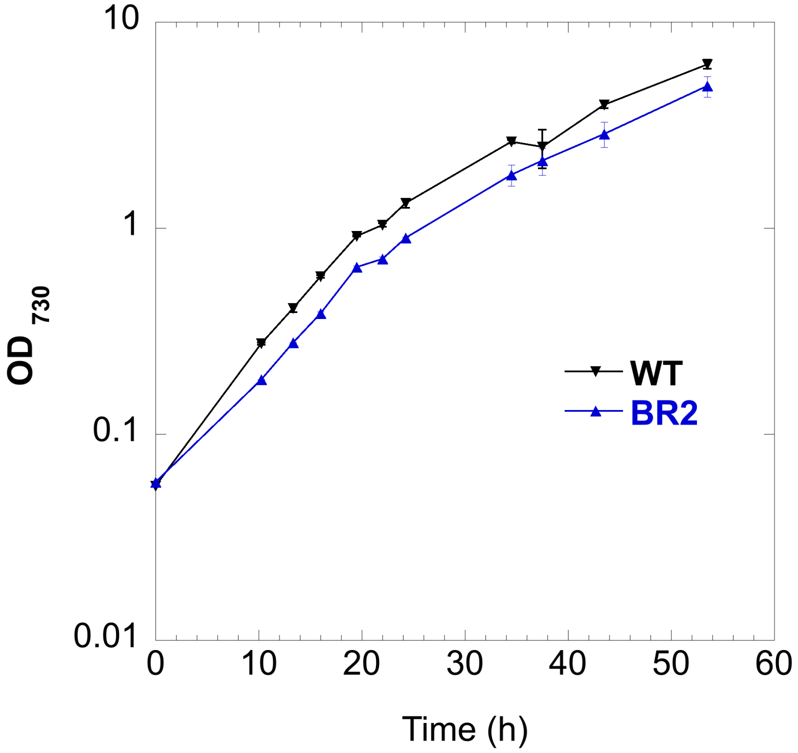

Supplement: Supplementary file 1 [file microorganisms-11-02593-s001.zip › Schluchter.et.al.Supplementary.materials.pdf]
